# Factors associated with primary healthcare provider access among trans and non-binary immigrants, refugees, and newcomers in Canada

**DOI:** 10.1016/j.jmh.2024.100241

**Published:** 2024-06-24

**Authors:** Monica A. Ghabrial, Tatiana Ferguson, Ayden I. Scheim, Noah J. Adams, Moomtaz Khatoon, Greta R. Bauer

**Affiliations:** a Epidemiology and Biostatistics, Schulich School of Medicine & Dentistry, Western University, London, ON, Canada; b Department of Psychology, Algoma University, Brampton, ON, Canada; c Black Queer Youth Collective, Toronto, ON, Canada; d Department of Epidemiology and Biostatistics, Dornsife School of Public Health, Drexel University, Philadelphia, PA, United States; e Unity Health Toronto, Toronto, ON, Canada; f Ontario Institute for Studies in Education, University of Toronto, Toronto, ON, Canada; g Center for Applied Transgender Studies, ON, Canada; h Transgender Professional Association for Transgender Health, ON, Canada; i Salaam Canada, Vancouver, BC, Canada; j PHS Community Services Society, Vancouver, BC, Canada; k Eli Coleman Institute for Sexual and Gender Health, Department of Family Medicine and Community Health, University of Minnesota Medical School, Minneapolis, MN, United States

**Keywords:** Transgender and non-binary health, Immigration, Refugees, Gender affirming care, Healthcare access, Healthcare provider

## Abstract

**Objective:**

Trans and non-binary (TNB) immigrants, refugees, and newcomers (IRN) face intersecting challenges and barriers, including stigma and persecution in countries of origin, and others unique to the Canadian resettlement process. The present study aimed to investigate factors that are associated with having a primary healthcare provider among TNB IRN.

**Design:**

Trans PULSE Canada was a community-based, national study of health and wellbeing among 2,873 TNB people residing in Canada, aged 14 and older, who were recruited using a multi-mode convenience sampling approach.. The survey asked questions about identity, community, service access, health – and IRN were asked questions specific to immigration/settlement.

**Results:**

Of the 313 IRN participants who completed the full survey version (age *M* = 34.1, *SE*=0.75), 76.4 % had a primary healthcare provider. TNB IRN largely reported being Canadian citizens (59.8 %), gender non-binary or similar (46.9 %), currently living in Ontario (35.5 %), and having immigrated from the United States (32.1 %). Chi-square analyses revealed that having a primary healthcare provider was associated with age, gender identity, citizenship status, region of origin, current location in Canada, length of time since immigrating to Canada, status in gender affirming medical care, and having extended health insurance. With modified Poisson regression, we found that TNB IRN who were non-permanent residents, originating from European, African, and Oceania regions, or living in Quebec and the Prairie provinces were less likely to have a primary healthcare provider.

**Conclusion:**

Results may inform settlement organizations of the unique needs and barriers of TNB IRN. Schools and LGBTQ+ organizations may better serve this population – especially those originating from highlighted regions, who live in Quebec or the Prairie provinces, and/or are non-permanent residents – by offering programs that connect them to primary healthcare providers who are competent in cross-cultural trans health.

## 1 Introduction

### 1.1 Healthcare barriers faced by trans and non-binary immigrants, refugees, and newcomers

As the immigrant, refugee, and newcomer (IRN) population in Canada grows, there remains limited research on the experiences and health outcomes of trans and non-binary (TNB) IRN. TNB IRN face unique intersecting healthcare challenges and barriers, which can include stigma and persecution in countries of origin and stressors unique to the process of resettling in Canada (Munro et al., 2013). The former may include transphobia, homophobia, and cissexism; the latter can additionally include delays in resettlement, discrimination in the immigration settlement process, socioeconomic difficulties, language barriers, isolation, and rejection from the larger LGBTQ+ (lesbian, gay, bisexual, trans, queer, and other sexual and gender minority identities) community due to racial and/or cultural discrimination (Castro et al., 2022). Qualitative research has shown that the Canadian refugee claims process causes emotional distress for LGBTQ+ people and that there are limited settlement resources to support this population (Kahn and Alessi, 2018), which may impact healthcare use. In addition to these psychosocial stressors, TNB IRN may not receive full benefit from the same factors that are often protective of cisgender and heterosexual migrant health – for example, the support of families and religious communities (Boulden, 2009).

Previous research has identified several factors that are associated with having a healthcare provider for the general population in Canada, as well as among IRN and TNB people more specifically, including age, race, education, province of residence, self-reported health (Degelman and Herman, 2016; Statistics Canada, 2020; Talbot et al., 2001), and rurality (Renner et al., 2021). Length of time since immigrating may also impact IRN healthcare use. According to a recent report from the 2019 Trans PULSE Canada study, TNB newcomers (those who immigrated within the last five years (Statistics Canada 2010)) were less likely to have a primary healthcare provider compared to established immigrants and individuals born in Canada (J. Navarro et al., 2021), which is in line with findings from earlier research with IRN in Canada (Asanin and Wilson, 2006). The Canadian Interim Federal Health Plan provides limited, temporary health-care coverage to people who are ineligible for provincial or territorial health insurance – in particular, protected persons, resettled refugees, and refugee claimants (Government of Canada, 2022). Individuals not covered by this plan and who are living in British Columbia, New Brunswick, Ontario, or Quebec, must wait three months before being eligible for a provincial health plan (e.g., Ontario Health Insurance Plan). During that waiting period, without substantial income or work health insurance, healthcare is largely inaccessible or unaffordable (Salami et al., 2020). Often, extended health services (e.g., most vision, dental, prescription drugs) are excluded from provincial health plans, which may further deter individuals without extended health insurance from seeking care (Asanin and Wilson, 2006).

Citizenship and immigration status have been noted in the literature as a critical barrier to health services among IRN (Sangaramoorthy and Guevara, 2017). TNB IRN without citizenship are less likely to possess legal documents that state their true gender, putting them at increased risk of discrimination, violence, and healthcare avoidance (Gyamerah et al., 2021). This may be exacerbated for IRN people of Color (Palazzolo et al., 2016). Refugee claimants in particular are less likely to pursue healthcare or have a healthcare provider due to fears of how their migration status may be affected (Campbell et al., 2014). Nevertheless, there is little research on the impact that immigration status may have on healthcare access among TNB IRN in Canada.

### 1.2 Intersectional and community-based research approach

Trans PULSE Canada was conceptualized and conducted to study health and wellbeing among TNB people, using an intersectional and community-based approach. Intersectionality, introduced by Black feminist scholars and activists (Crenshaw, 1989; Combahee River Collective 1977), is an analytical framework that can be used to describe and examine how interlocking power systems (e.g., racism, cisheterosexism) impact lived experience and cause health inequities (Agénor, 2020). With objectives that include social justice (e.g., contributing to policies and programs developed for health equity), effective intersectional research requires an understanding of community politics and strong relationships with community stakeholders, making it compatible with a community-based approach (Agénor, 2020; Fernández-Sánchez, 2023). In community-based research, community members and organizations are equitably engaged, collaborating with academic researchers in all phases of research from design to dissemination (Strand et al., 2003). The Trans PULSE Canada study was designed for both intra- and inter-categorical examination. We thus collaborated with 9 Priority Population Teams belonging to multiply marginalized communities (including trans and non-binary people who are: Indigenous, Gender Non-binary, Racialized as People of Color, Sex Workers, Older Adults, Youth, Northern and/or Rurally Living, Disabled, and Immigrants, Refugees, and/or Newcomers). Through this process, the IRN Priority Population group operationalized IRN categorization and had decision-making power to add survey content and request questions.

### 1.3 The present study

The present study involved a sub-analysis with Trans PULSE Canada respondents who reported being immigrants, refugees, or newcomers. The objectives of this article are to describe the characteristics of TNB IRN in Canada, to determine the proportion that have a primary healthcare provider, and to explore if having a primary healthcare provider is associated with sociodemographic characteristics, migration experiences, and citizenship status in our sample.

## 2 Material and methods

### 2.1 Sample and procedure

Full-survey data used in the present analysis were collected from July 26 to October 1, 2019 from Canadian residents aged 14 and older, who were recruited using a multi-mode convenience sampling approach. The survey could be completed in English or French online, by mail, by telephone, or in-person in 11 major urban areas. Interpretation into any language other than English or French was available via telephone (no participants selected this option). The survey included skip logic, wherein only participants who identified with certain subpopulations (e.g., IRN) were prompted to complete survey items relevant to those groups. Trans PULSE Canada was promoted on social media, email listservs, at community agencies and events targeted toward sexual and gender minority people, and through peer research associate outreach. Participants were given the option of completing the full (∼60-minutes) or short-form (∼10 min) survey. Trans PULSE Canada was approved by the research ethics boards of Western University, Unity Health Toronto, Wilfrid Laurier University and the University of Victoria.

### 2.2 Measures

#### 2.2.1 Sociodemographic characteristics

All participants were asked questions regarding social and demographic characteristics, including age, sex assigned at birth, gender, province, and details related to educational background, employment, and income. Participants were asked to select their ethnoracial background from a provided list (or write-in text option). All who selected an ethnoracial minority background label designated as a visible minority by the Government of Canada (Statistics Canada, 2021) were coded as an ethnoracial minority. Participants selected one of four gender identity groups, which were outlined by community member collaborators: man or boy; woman or girl; Indigenous or other cultural gender minority identity (e.g., Two Spirit, Hijra); non-binary, genderqueer or similar. Due to small numbers, we grouped individuals identifying as Indigenous or other cultural gender minority identity with the non-binary category for analyses.

Current Canadian province or territory was collapsed into five categories: Prairie Provinces (Manitoba, Saskatchewan, Alberta); Quebec; Ontario; British Columbia; and Northern Territories and Atlantic Region (New Brunswick, Newfoundland and Labrador, Nova Scotia). For analyses, Northern Territories and Atlantic Region were combined due to small numbers.

Participants selected household income from a list of 12 categories (e.g., less than $10,000, $10,000 to less than $15,000, $20,000 to less than $30,000). We estimated numerical income by assigning the mid-point of the participant's selected category (e.g., for the category $60,000 to less than $80,000, participants were assigned the amount of $70,000). Participants who selected the lowest or highest income category (less than $10,000; $150,000 or more) were assigned $9999 and $150,000, respectively. Participants also reported the number of dependents supported with the household salary. We compared income and number of supported dependents to the Statistics Canada low-income measure (Statistics Canada, 2021) and participants were categorized dichotomously based on whether or not they were below this low-income cut-off (Yes/No).

#### 2.2.2 Immigration-related variables

IRN participants reported the length of time since they arrived in Canada (in months and/or years) and their country of origin. Country of origin was condensed into seven regions: United States, Latin America and the Caribbean; Northern and Sub-Saharan Africa; Central, Eastern, Southern, South-Eastern, Western Asia; Eastern, Northern, Southern, Western Europe; and Oceania.

Using a provided list (e.g., education, gender-affirming health care, persecution as a trans or non-binary person, intimate partner violence), IRN respondents selected all applicable reasons for migrating. For our primary analysis, those who endorsed any healthcare-related reason (with or without other reasons) were categorized as such. Healthcare-related reasons included immigrating for gender-affirming care, other healthcare for self, or healthcare for another person.

Respondents also reported their current citizenship status in Canada from a provided list, which we grouped using Immigration, Refugee, and Citizenship Canada categories (i.e., Naturalized citizen, permanent resident, and non-permanent resident). Participants were categorized as a non-permanent resident unless they identified as either a permanent resident or a Canadian citizen. Non-permanent resident options included visitor, student (e.g., study permit), and work permit (e.g., temporary foreign worker).

Participants were asked if they accessed any settlement services within their first 12 months in Canada. They selected responses from a provided list that included immigration lawyer or consultant, settlement organization, language training, other education, and LGBTQ+, faith-based, or region-of-origin-related organizations and groups. We categorized participants into two groups: those who accessed one or more of these services and those who did not.

#### 2.2.3 Status in gender affirming care

Participants selected one statement from a list of 5 that best described their current situation regarding puberty blockers, hormones, and/or surgery (e.g., “I am in the process of completing gender-affirming medical treatment,” “I am not sure whether I am going to seek gender-affirming medical treatment”). We grouped these responses into three categories: 1) have had wanted/needed care; 2) in process or planning to, but not begun; 3) unsure or not planning to pursue.

#### 2.2.4 Anticipated discrimination

The Intersectional Discrimination Index – Anticipated (InDI-A) consists of nine items assessing anticipated discrimination on the basis of any social identity or position (Scheim and Bauer, 2019). Participants rank their agreement with each statement on a scale from 0 (*Strongly disagree*) to 4 (*Strongly agree*) and the scores are averaged. Examples of items include: “Because of who I am, I might have trouble finding or keeping a job”; “I worry about being harassed or stopped by police or security”; “I expect to be pointed at, called names, or harassed when in public”. Internal consistency in the original study was good (*a* = 0.93; Scheim and Bauer, 2019), as was that in the present sample (*a* = 0.87).

#### 2.2.5 Access to a primary healthcare provider

Participants were asked: “Do you currently have a primary health care provider? By this, we mean one health professional that you regularly see or talk to when you need care or advice for your health.” Response options included: *Yes, a family doctor; Yes, a nurse practitioner; No, I receive primary health care at a walk-in clinic; Not at the present time.* To compute a dichotomous variable, participants who reported having a family doctor or nurse practitioner were coded as Yes and those who selected either of the latter two options were coded as No.

## 3 Analysis

### 3.1 Missing data

Data for the variables used in the present analyses were complete for 52.1 % of the TNB IRN participants who completed the full version of the survey. Missingness in variables used in this analyses ranged from 0 to 19.8 %. The measure missing the most data (19.8 %) was InDI-A, which could likely be attributed to the fact that it was located near the end of the survey. Apart from the InDI-A, the range of missingness for variables of interest was 0 to 13.4 %. We assessed the pattern of all missing data included in this analysis and determined data to be missing at random. To avoid participant loss in complete case analysis, we used multiple imputation with fully conditional specification. Fully conditional specification was deemed appropriate for the present study, as this is a powerful and statistically valid approach to impute for large datasets that contain both categorical and continuous variables (Liu and De, 2015). For modified Poisson regression analyses, we produced twenty imputations and weighted regression analyses after imputation. The outcome variable was not imputed.

### 3.2 Survey weights and descriptive analyses

Our analyses used full survey data that were weighted to apply to the demographic distribution of the larger sample who completed either the full or short-form survey. Weights were produced using Stata ipfweight, which uses iterative proportional fitting to perform stepwise adjustment to achieve known population margins.

We produced chi-square tests of independence to examine the associations between our outcome (has a primary healthcare provider: yes/no) and age category, gender, sex assigned at birth, ethnoracial minority category (yes/no), Statistics Canada Low-income category (yes/no), rurally living (yes/no), highest level of education, citizenship status, reasons for migrating (healthcare-related/not healthcare-related), region of origin, current location in Canada, length of time since immigrating to Canada, use of settlement services within first 12 months (yes/no), self-reported health, status in gender affirming care, and prescription insurance (yes/no). We used an independent samples *t*-test to examine the relationship between our outcome and anticipated discrimination.

### 3.3 Modified Poisson regression

Modified Poisson regression (Zou, 2004) was used to investigate if having a primary healthcare provider was associated with gender, ethnoracial background, citizenship status, reasons for migrating, region of origin, current location in Canada, length of time since migrating (in number of months), use of settlement services within first 12 months of migrating, and status in gender affirming care before and after accounting for potential mediating effects of anticipated discrimination. Based on community knowledge and information from the existing literature (e.g., Degelman and Herman, 2016; Talbot et al., 2001; Renner et al., 2021; Asanin and Wilson, 2006; Statistics Canada 2016), we controlled for age, sex assigned at birth, rurality, highest level of education, self-reported health, and whether or not participants had extended health insurance that covered prescription costs.

Using parameter estimate output, we calculated the final relative risks in Microsoft Excel. All other analyses were conducted with SAS 9.4 (SAS 9.4 2013). Our initial model contained our predictor variables and covariates. In the second model, we entered anticipated discrimination, exploring potential mediating effects. Finally, to examine if citizenship status would moderate the effects of gender and race on primary healthcare provider access, we examined a third model in which we entered interaction terms (gender x citizenship status; ethnoracial background x citizenship status).

## 4 Results

### 4.1 Sample characteristics and characteristics of individuals with a primary healthcare provider

The age range of the 313 TNB IRN in this study was 14 to 74 years (age *M* = 34.1, *SE*=0.75). The average length of time that participants had lived in Canada was 16.9 years (*SE*=13.2) and ranged from 1 month to 68 years. The average age of immigration was 19.2 (*SE*=0.85) and ranged from 8 months to 52.8 years. Nine (3.2 %) participants reported being a refugee or protected person, an asylum or refugee claimant, or in Canada on humanitarian and compassionate grounds. 76.4 % of participants had a primary health care provider. The most commonly reported immigration service used was school (31.4 %), followed by LGBTQ organizations (24.7 %), lawyers (13.9 %), settlement organizations (9.6 %), faith-based organizations (9.2 %), language training (7.9 %), LGBTQ immigrant and settlement organizations (6.9 %), and organizations or community groups from country of origin (5.1 %). See Table 1 for frequencies and weighted proportions of sociodemographic characteristics and weighted proportions of having a primary healthcare provider stratified by sociodemographic categories, immigration-related variables, and health-related variables. Table 1 also presents results from chi-square tests of independence, examining the differences between categories in weighted proportion of having a primary healthcare provider. All reasons for migrating that were selected by respondents from the provided list can be found in Table 2.

**Table 1:** Sociodemographic characteristics, weighted proportions of having a healthcare provider, and unadjusted comparisons.

	Sociodemographic characteristics	n	Weighted Percent (95 % CI)	Has Healthcare Provider; Weighted Percent	95 % CI	P-value^a^
Age	14–18	13	4.2 (1.9, 6.4)	72.7	46.1, 99.3	.0003⁎⁎
	18–25	72	23.3 (18.5, 28.0)	61.1	49.1, 73.2	
	26–49	180	57.5 (52.0, 63.1)	77.5	71.1, 84.0	
	50+	48	15.1 (11.1, 19.0)	97.5	92.8, 100.0	
Gender^b^	Man/boy	66	21.9 (17.2, 26.6)	80.8	70.0, 91.5	<0.0101*^,^^c^
	Woman/girl	89	30.2 (24.9, 35.5)	86.2	78.8, 93.5	
	Indigenous or other cultural gender minority identity	3	1.1 (0.0, 2.4)	60.2	0.0, 100.0	
	Non-binary or similar	144	46.9 (41.2, 52.6)	68.7	60.9, 76.5	
Sex assigned at birth	Male	117	39.6 (34.0, 45.2)	77.39	69.5, 85.3	.7731
	Female	184	60.4 (54.8, 66.0)	75.88	69.3, 82.5	
Racialized as a person of Color (self-report)	Yes	107	36.0 (30.5, 41.4)	78.0	(69.4, 81.8)	.6629
	No	206	64.0 (58.6, 69.5)	75.6	(69.3, 86.6)	
Ethnoracial minority or Indigenous background d	Yes	127	42.4 (36.8, 47.9)	75.5	67.4, 83.7	.7721
	No	186	57.6 (52.1, 63.2)	77.1	70.7, 83.4	
Selected Ethnoracial background e	Black African	14	5.4 (2.6, 8.1)			
	Black Canadian or African American	4	1.6 (0.0, 3.3)			
	Black Caribbean	6	2.2 (0.4, 4.0)			
	East Asian	23	7.7 (4.6, 10.7)			
	Indigenous	14	4.4 (2.2, 6.7)			
	Indo-Caribbean	7	2.6 (0.7, 4.5)			
	Latin American	39	12.5 (8.8, 16.1)			
	Middle Eastern / North African	12	4.1 (1.8, 6.4)			
	South Asian	22	7.2 (4.3, 10.1)			
	Southeast Asian	15	5.1 (2.6, 7.7)			
	White Can or Am	115	35.9 (30.5, 41.2)			
	White European	127	39.7 (34.2, 45.2)			
	Other	17	5.7 (3.0, 8.4)			
Low Income (Statistics Canada measure)	Yes	165	57.6 (51.7 63.4)	82.3	76.1, 88.6	.0751
	No	118	42.5 (36.6, 48.3)	72.9	64.4, 81.5	
Rurally living	Yes	20	6.5 (3.7, 9.2)	81.4	62.4, 100.0	.6524
	No	283	93.6 (90.8, 96.3)	76.5	71.3, 81.8	
Education	Less than high school	23	7.4 (4.4, 10.3)	66.0	45.2, 86.8	.7247
	High school or equivalent	24	7.7 (4.7, 10.7)	73.5	55.1, 91.9	
	Some college or university	68	21.6 (17.0, 26.1)	73.9	62.4, 85.4	
	Complete college/university	128	41.4 (35.9, 46.9)	78.7	71.0, 86.4	
	Graduate degree	70	22.0 (17.4, 26.6)	78.8	68.9, 88.7	
Citizenship status in Canada	Permanent resident	76	24.2 (19.4, 29.0)	75.6	65.7, 85.5	<0.0001⁎⁎
	Canadian citizen	187	59.8 (54.4, 65.3)	84.9	79.3, 90.6	
	Non-permanent resident	50	16.0 (11.9, 20.0)	47.4	32.9, 61.9	
Reasons for migrating	Healthcare related	54	17.3 (13.1, 21.6)	75.0	63.5, 86.5	.7848
	Non-healthcare related	259	82.7 (78.4, 86.9)	76.8	71.2, 82.4	
Region/country of origin	Latin America and the Caribbean	35	11.9 (8.1, 15.6)	89.5	78.0, 100.0	.0034⁎⁎
	Northern or Sub-Saharan Africa	19	6.5 (3.6, 9.4)	55.4	31.2, 79.6	
	Central, Eastern, Southern, South-Eastern, Western Asia	46	15.5 (11.3, 19.7)	78.4	65.7, 91.0	
	Eastern, Northern, Southern, Western Europe	96	30.3 (25.2, 35.5)	71.73	61.9, 81.6	
	Oceania	12	3.7 (1.6, 5.8)	41.4	13.4, 69.4	
	United States	101	32.1 (26.8, 37.3)	82.9	75.4, 90.4	
Current Location in Canada	Quebec	32	10.6 (7.1, 14.1)	42.6	24.1, 61.1	<0.0001**f
	Prairie Provinces	53	17.2 (12.9, 21.4)	70.8	57.3, 84.3	
	Alberta	44	14.4 (10.4, 18.3)	—	—	
	Manitoba	3	.94 (0.00, 2.00)	—	—	
	Saskatchewan	6	1.9 (0.4, 3.4)	—	—	
	British Columbia	102	32.2 (27.0, 37.4)	78.4	70.1, 86.7	
	North Region and Atlantic Region	14	4.5 (2.2, 6.8)	75.6	51.2, 99.9	
	Northwest Territories, Nunavut, Yukon	2	.64 (0.00, 1.53)	—	—	
	New Brunswick	6	2.0 (0.4, 3.6)	—	—	
	Newfoundland & Labrador	1	.30 (0.00, 0.89)	—	—	
	Nova Scotia	4	1.3 (0.0, 2.5)	—	—	
	Prince Edward Island	1	.30 (0.00, 0.89)	—	—	
	Ontario	110	35.5 (30.1, 40.9)	86.9	80.2, 93.6	
Length of time since immigrating to Canada	Less than 1 year	130	41.7 (36.2, 47.3)	76.4	67.9, 84.8	.0002⁎⁎
	1 to less than 3 years	27	8.6 (5.5,11.8)	47.8	28.9, 66.8	
	3 to less than 5 years	17	5.3 (2.8,7.8)	64.4	41.5, 87.4	
	5 to less than 10 years	24	7.7 (4.7,10.6)	79.7	63.7, 95.8	
	10+ years	115	36.7 (31.3,42.1)	84.5	77.6, 91.3	
Used any settlement services within first 12 months	Yes	145	50.5 (44.7, 56.4)	74.6	67.4, 81.7	.4603
	No	143	49.5 (43.6, 55.3)	78.4	71.3, 85.4	
Self-reported health	Excellent or very good	127	45.1 (39.2, 51.0)	77.0	69.6, 84.4	.9595
	Good	89	31.3 (25.8, 36.7)	76.6	67.7, 85.5	
	Poor	67	23.6 (18.6, 28.6)	75.1	64.4, 85.9	
Gender affirming care status	Have had care	77	28.6 (23.1, 34.0)	89.2	81.9, 96.3	<0.0001⁎⁎
	In process or planning to begin	108	40.1 (34.2, 46.0)	80.4	73.0, 87.9	
	Unsure or not planning to pursue	86	31.3 (25.8, 36.9)	59.0	48.4, 69.6	
Insurance covers most or all prescriptions	Yes	185	58.4 (52.9, 64.0)	83.4	78.0, 88.8	.0003⁎⁎
	No	128	41.6 (36.0, 47.1)	63.4	53.6, 73.2	
Anticipated Discrimination *M (SE)*		2.36 (0.05)		2.34 (0.06)	2.22, 2.45	

Note:.

CI, Confidence interval; InDI-A, Intersectional Discrimination Index – Anticipated Discrimination; M, mean; SE, standard error.

a P values from chi square tests of differences between groups in having a healthcare provider.

b Non-binary gender category includes all gender identities that did not fit within binary gender categories.

c Non-binary or similar gender is grouped with Indigenous or other cultural gender minority identities for chi square analysis.

d Ethnoracial background category was determined by the authorship team to include all individuals who did not identify as exclusively White European or White Canadian. This is in line with the Employment Equity Act of Canada.

e Participants selected all ethnoracial background labels that applied. These selections were used in the “Ethnoracial minority or Indigenous background” groupings.

f Analysis compares regions of Canada: Prairie Provinces (Manitoba, Saskatchewan, Alberta), British Columbia, and Atlantic Region (New Brunswick, Newfoundland and Labrador, Nova Scotia) and Northern Territories.

⁎ *p* < .05.

⁎⁎ *p* < .01.

**Table 2:** Reasons for migrating to Canada (select all that apply).

Reasons	Frequency	Weighted %
Employment/labour	101	34.9
Education or training	93	32.2
Living conditions	95	33.1
Gender affirming care for self	34	12.1
Healthcare for self	30	10.6
Healthcare for family	15	5.1
Lifestyle change or for enjoyment	67	23.1
Escape sociopolitical conditions in home country	50	17.7
Trans or non-binary persecution	36	13.3
Persecution based on sexual orientation	27	10.1
Religious persecution	11	3.9
Conditions of war, slavery, or forced labour	10	3.6
Domestic violence/intimate partner violence	13	4.6
Family reasons	107	36.9
Visitor/tourist	5	1.6
Other	14	4.9
Unsure	7	2.5

We found a significant relationship between having a primary healthcare provider and age, χ^2^(3) = 18.60, *p* = .0003, gender, χ^2^(2) = 9.19, *p*=.0101, citizenship status, χ^2^(2)= 27.89, *p*<.0001, region of origin, χ^2^(5) = 18.04, *p*=.0034, current location in Canada, χ^2^(4) = 23.92, *p*<.0001, length of time since immigrating to Canada, χ^2^(4) = 22.37, *p*=.0002, current situation in gender affirming care, χ^2^(2) = 20.69, *p*<.0001, and whether or not the respondent had extended insurance that covers prescriptions, χ^2^(1) = 12.97, *p*=.0003. There were no statistically significant relationships between having a primary healthcare provider and ethnoracial background, sex assigned at birth, low-income category, living rurally, education, reasons for migrating (healthcare-related or not), use of settlement services, self-reported health, nor anticipated discrimination, *t*(248)=0.40, *p*=.5271.

### 4.2 Factors predicting having a primary healthcare provider

In the initial regression model (Table 3), we found that significant predictors included citizenship status, region of origin, current location in Canada, and status of gender affirming care. Specifically, we found that participants who were Canadian citizens (RR=1.35 95 % CI 1.05, 1.73) and permanent residents (RR=1.28, 95 % CI 1.01, 1.62) were significantly more likely to have a primary healthcare provider than non-permanent residents (reference category). Concerning region of origin, TNB IRN from Northern or Sub-Saharan Africa (RR=0.72, 95 % CI 0.54, 0.96), Eastern, Northern, Southern, or Western Europe (RR= 0.81, 95 % CI 0.67, 0.94), and Oceania (RR=0.55, 95 % CI 0.32, 0.94) were significantly less likely to have a primary healthcare provider compared to those from the United States. Compared to TNB IRN in Ontario, participants in Quebec (RR = 0.70, 95 % CI 0.53, 0.92) and the Prairie provinces (RR = 0.84, 95 % CI 0.71, 0.99) were significantly less likely to have a primarily healthcare provider. Compared TNB IRN in Ontario, we found smaller proportions in British Columbia (RR = 0.88, 95 % CI 0.78, 1.00) and North and Atlantic Regions (RR = 0.76, 95 % CI 0.55, 1.04). Individuals who were not planning to pursue gender affirming medical care or were unsure about their plans were significantly less likely to have a primary healthcare provider compared to those planning to pursue or already in process (RR = 1.34, 95 % CI 1.05, 1.73) and those who have had the care that they need/want (RR = 1.24, 95 % CI 1.01, 1.73).

**Table 3:** Modified Poisson regression models predicting having a primary healthcare provider among transgender and non-binary immigrants, refugees, and newcomers in Canada.

	Model 1		Model 2	
Variable	Relative Risk	95 % CI	Relative Risk	95 % CI
Gender				
Man or boy	1.00	.86, 1.17	.99	.85, 1.14
Woman or girl	.94	.76, 1.16	.94	.76, 1.17
Nonbinary or similar, or Indigenous or other cultural gender identity	ref	ref	ref	ref
Ethnoracial minority or Indigenous background				
Yes	.98	.85, 1.13	.98	.85, 1.14
No	ref	ref	ref	ref
Status				
Permanent resident	1.28*	1.01, 1.62	1.29*	1.02, 1.64
Canadian citizen (naturalized)	1.35*	1.05, 1.73	1.35*	1.05, 1.73
Non-permanent resident	ref			
Reasons for migrating				
Healthcare related	.89	.76, 1.04	.89	.76, 1.04
Non healthcare related	ref	ref	ref	ref
Region of origin				
Latin America and the Caribbean	1.00	.82, 1.21	1.00	.82, 1.21
Northern & Sub-Saharan Africa	.72*	.54, 0.96	.73*	.55, 0.97
Central, Eastern, Southern, South-Eastern, Western Asia	.93	.77, 1.12	.94	.78, 1.13
Eastern, Northern, Southern & Western Europe	.81⁎⁎	.67, 0.94	.80⁎⁎	.69, 0.93
Oceania	.55*	.32, 0.94	.55*	.32, 0.95
United States	ref	ref	ref	ref
Current location in Canada				
Quebec	.70*	.53, 0.92	.69*	.52, 0.91
Prairie Provinces	.84*	.71, 0.99	.83⁎⁎	.70, 0.99
British Columbia	.88	.78, 1.00	.88	.78, 1.00
North Region and Atlantic Provinces	.76	.55, 1.04	.76	.55, 1.05
Ontario	ref	ref	ref	ref
Length of time in Canada	1.00	1.00, 1.00	1.00	1.00, 1.00
Accessed settlement services	1.05	.91, 1.21	1.05	.99, 1.21
Gender-affirming care status				
Have had care	1.24*	1.05, 1.46	1.25⁎⁎	1.06, 1.46
In process, or planning to but not begun	1.34⁎⁎	1.12, 1.59	1.34⁎⁎	1.13, 1.60
Unsure or not planning to receive	ref	ref	ref	ref
Anticipated discrimination	–	–	.99	.89, 1.04

⁎ *p* < .05.

⁎⁎ *p* < .01 Note: Adjusted for age, education, sex assigned at birth, self-reported health, prescription insurance, rurality Model 3, not shown, had non-significant interactions, which were gender x citizenship status; ethnoracial background x citizenship status Ref, reference group.

In the second model, anticipated discrimination did not contribute significantly (RR=0.96, 95 % CI 0.89, 1.04). Including this variable in the model did not alter the statistical significance or direction of the effects of the other variables, indicating no mediating effect.

Finally, neither of the interaction terms (gender x citizenship status; ethnoracial background x citizenship status) were statistically significant, suggesting that the effects of citizenship status did not vary by gender nor by ethnoracial background, when controlling for other variables in the model.

## 5 Discussion

The present study provides novel information about the characteristics of TNB IRN in Canada, their use of immigration and settlement services, and the potential structural factors that may reduce their access to a primary healthcare provider. The two most commonly used services were schools and LGBTQ organizations, which highlights the need to equip these settings with knowledge and resources to better serve TNB IRN. Analyses revealed that TNB IRN who were non-permanent residents, originating from Northern or Sub-Saharan Africa, Eastern, Northern, Southern, or Western Europe, or Oceania currently located in Quebec or the Prairie provinces, and/or who were not pursuing gender affirming care were at the greatest risk of not having a primary healthcare provider.

Although the majority (76.4 %) of our sample reported having a primary healthcare provider, this number was still less than the proportion for Canadian-born respondents in Trans PULSE Canada (81 %; (J. Navarro et al., 2021)), the proportion of immigrants and newcomers not identifying as TNB (84 %, (Ravichandiran et al., 2022)), and the proportion in the general Canadian population (86 %; (Statistics Canada 2022)). There is universal health coverage in Canada, yet many barriers exist for TNB IRN, as well as Canadian-born citizens, including a severe physician shortage throughout Canada (Malko and Huckfeldt, 2017). Healthcare barriers faced by TNB IRN include language barriers and lack of cultural competence, transphobia and lack of trans health knowledge, xenophobia (Safer et al., 2016), denial of care (Bauer et al., 2014), refusal to approve gender affirming medical care (Rotondi et al., 2013), and the intersecting impact of these factors – all of which may reduce the likelihood for TNB IRN to have a primary healthcare provider.

Frequencies of having a primary healthcare provider varied greatly by province. Proportions were higher in Ontario compared to all other provinces and regions – especially Quebec and the Prairie Provinces. The proportion of our survey respondents in Quebec who had a primary healthcare provider was the lowest, which is consistent with patterns found among immigrants (Setia et al., 2011) and the general Canadian population (Statistics Canada 2020). However, the proportion of TNB IRN in Quebec from our study with a primary healthcare provider (∼43 %) is still markedly lower than rates found in the general population of Quebec (78.5 %; (Statistics Canada 2020)). Additionally, proportions in Quebec and the Prairie provinces were lower in our study compared to numbers found in the general TNB population (Scheim et al., 2021). This suggests that there are possible immigration-related factors in these areas that further bar TNB IRN from healthcare use beyond factors already faced by Canadian-born TNB. Previous research has suggested that newcomers with precarious immigration status and acute health crises residing in Quebec may struggle to navigate the health system (Kuile et al., 2007). In 2022, Bill 96 was established in Quebec, which enforces the use of French in businesses and public services. Scholars and reporters speculate that this new law may deter IRN from pursuing services and healthcare (Stevenson, 2022; Polese, 2022). Although Bill 96 was not in effect at the time of data collection, the consequences may provide some insight about language as a barrier to healthcare in Quebec for TNB IRN, who may struggle to confidently communicate their needs – and general trans health matters – in this setting.

Respondents identifying as men or women were more likely to have a primary healthcare provider than those who identified as non-binary or with an Indigenous or other cultural gender minority identity. Medical practice has a long history of enforcing a gender binary (Vincent, 2020), which may deter non-binary individuals from pursuing healthcare (Paine, 2018). These findings are in line with results from an earlier Trans PULSE Canada report showing that non-binary people are less likely to have a primary healthcare provider than other study participants (J Navarro et al., 2021) and evidence that non-binary patients experience higher rates of disrespect from healthcare providers than binary trans patients (Kattari et al., 2020). We also found an association between having a primary healthcare provider and age, wherein young adults (ages 18–25) were the least likely to report having a healthcare provider. These results are somewhat reflective of reports from the general Canadian population, showing that youth aged 12 to 17 are the least likely age group to have seen a physician in the last year (59.7 %), followed by those aged 18 to 34 (64 %; (Statistics Canada 2022)). These findings highlight a potential need for targeted services for young adult TNB IRN.

When examining length of time since arriving in Canada, we found that individuals who had arrived one to three years prior to data collection reported the lowest proportion of having a primary healthcare provider (∼ 48 %), followed by three to five years (∼64 %). Meanwhile, the proportion of respondents who had been in Canada for under one year and had a primary healthcare provider was relatively high (∼76 %) and similar to that of established immigrants (∼80–85 %). Past research with IRN in Canada has shown that established immigrants (commonly defined as having been in Canada for 10+ years) are more likely to report having a healthcare provider than newcomers (e.g., Degelman and Herman, 2016; Ravichandiran et al., 2022). These findings may be in line with recent trends in immigration patterns and policy, projecting an increase in immigrants who are younger and more highly educated (Ravichandiran et al., 2022), and with legislative changes that favour immigrants who are deemed more likely to contribute to Canada's economy (Fuller-Thomson et al., 2011). In other words, IRN in our study who have been in Canada fewer than 12 months may possess more resources to assist in accessing a primary healthcare provider than IRN who have been in Canada one to three years. Alternatively, this pattern may reflect the *Healthy Immigrant Effect*, in which immigrants at first exhibit better health outcomes than domestic-born populations, but decline in health over time due to assimilation-based mental health effects, wear and tear of stressors, and reduced healthy behaviors (Elshahat et al., 2022). It is possible that one of the health behaviours impacted by the *Healthy Immigrant Effect* is having a primary healthcare provider.

Our regression results revealed that citizenship status contributed to having a primary healthcare provider, wherein individuals who were naturalized citizens and permanent residents were more likely to have a primary healthcare provider than non-permanent residents. Citizenship and length of time in Canada are highly related; permanent residents who have lived in Canada for three out of the most recent five years are eligible to apply for citizenship (Government of Canada, 2019). However, there are variations and exceptions. For example, government assisted refugees are sponsored as permanent residents upon arrival in Canada (Immigration, Refugees and Citizenship Canada, 2019). Additionally, individuals may reside in Canada for several years with student or work visas without applying for permanent residence, and students must work for an additional period of time after graduating to qualify (Government of Canada, 2022). Despite universal healthcare in Canada, there are multiple circumstances in which non-permanent residents may not have access to healthcare services, including the noted four-month wait in British Columbia, New Brunswick, Ontario, and Quebec. Refugee claimants who are appealing a rejection lose their eligibility for public insurance, including Interim Federal Health Benefits (Caulford and Vali, 2006). Additionally, healthcare access and coverage may vary for non-permanent residents. For example, students can purchase healthcare through their university and may access it via on-campus clinics without needing a primary healthcare provider, and individuals on work visas can apply for provincial coverage only with proof of full-time, permanent employment.

Region of origin (e.g., Northern or Sub-Saharan Africa, Eastern, Northern, Southern, or Western Europe, and Oceania) may also be a key factor in having a primary healthcare provider for IRN in Canada. The existing literature suggests that people from certain regions, cultures, and ethnoracial backgrounds are less likely to have a doctor because they prioritize gender, language, and cultural similarities and relatability in their physician (Talbot et al., 2001; Vu et al., 2016) – which has been substantiated in research with Canadian immigrants (Kalich et al., 2016; Ahmed et al., 2016). Relatedly, cross-cultural communication skills and awareness in tactfully addressing sensitive subject matter has been highlighted as a deterrent for immigrants accessing care in Canada (Ahmed et al., 2016). In the present study, it may be that respondents originating from certain regions have struggled to find physicians that meet these criteria, while those from other regions (e.g., East Asia, the United States) had greater ease in locating physicians that share or have a similar cultural background or that speak the same language. For example, in 2018, 17.1 % of physicians in the United States were Asian, while only 5 % were Black or African American (Association of American Medical Colleges, 2019). These distributions appear to be similar to ethnoracial background representation in Canadian medical schools, with 11.2 % and 8.8 % of medical students identifying as Chinese and South Asian, respectively, but only 1.7 % identifying as Black (Khan et al., 2020). This is complicated by the fact that few physicians are competent in TNB health, regardless of cultural, ethnoracial, or immigration background, making finding a suitable physician particularly challenging. Cultural stigma of certain diseases and health concerns (e.g., depression) may also present a barrier to seeking care (Kahn and Alessi, 2018; Kalich et al., 2016). Yet, these factors do not necessarily address why individuals immigrating from the Oceania region (primarily Australia) also reported low rates of access to primary healthcare. Future research is therefore needed to investigate these differences.

Status or history of gender affirming care contributed significantly to our predictions of having a primary healthcare provider, revealing that individuals who were planning to pursue, in process, or have had gender affirming care were more likely to have a primary healthcare provider than those who were unsure or not planning to pursue such care. Our findings suggest that gender affirming care is a stronger driver of having a physician than health-related reasons for immigrating. Anticipated discrimination did not appear to predict our outcome, nor mediate any relationships in our model. Here, the distinction between having a healthcare provider and visiting a healthcare provider may be relevant. Previous research with transgender and IRN populations, respectively, has shown that anticipated discrimination is associated with healthcare avoidance (Khan et al., 2020; Pollock et al., 2012). It may be that participants who anticipate discrimination have a primary healthcare provider that they nevertheless avoid due to anticipated and/or experienced discrimination at the clinic (e.g., Pollock et al., 2012).

### 5.1 Limitations

As we used convenience sampling, our sample may not represent the wider population of TNB IRN – for example, around the matter of language. Respondents to the Trans PULSE Canada study were given the option to complete the survey in languages other than French or English via a telephone translator; however, none selected this option. We were thus unable to assess language as a factor in having a primary healthcare provider from individuals who do not have command of Canada's official languages. Due to the size of our sample, we pooled all individuals that might be categorized as an ethnoracial minority in Canada. Doing so may have washed out inequalities experienced by particular ethnoracial minority groups. Relatedly, we grouped regions in Canada together due to small sample sizes in some provinces and territories. This limits our understanding of potential inter-provincial variation. Finally, analyses were cross-sectional and thus temporality and causality cannot be determined.

### 5.2 Conclusions and implications

This study was a novel examination of factors associated with having a primary healthcare provider for TNB IRN in Canada. Our findings suggest that citizenship, region of origin, current location in Canada, and status in gender affirming care may be key considerations in determining healthcare use and access. Our results revealed that non-permanent residents may face more barriers in obtaining a primary healthcare provider compared to permanent residents and naturalized citizens. The dramatically low proportion of TNB IRN participants with a primary healthcare provider in Quebec is especially alarming in light of the fact that Quebec is one of the top three destinations in Canada for IRN (Statistics Canada 2016). Research is needed which investigates specific healthcare barriers for TNB IRN in Quebec. Our study highlights regions of origin whose migrants may benefit from additional services and resources that aid in navigating the Canadian healthcare system. Results suggest a need for physicians knowledgeable in TNB health who belong to these specific cultural communities or are familiar with cultural factors relevant to varied communities and who have access to translation services that are likewise knowledgeable in TNB health. Further research is needed to explore differences in healthcare use across and within multiethnic and multilinguistic TNB IRN communities. Our results revealed that schools and LGBTQ+ organizations are among settlement organizations most commonly used, suggesting that these settings in particular may need specific resources in connecting TNB IRN with a primary healthcare provider.

Access to safe and competent care is critical to mental and physical health. TNB patients who are disrespected by or tasked with educating their primary healthcare provider are more likely to experience depression and suicidal thoughts (Kattari et al., 2020). The stage that an individual is at in the pursuit of gender affirming care was determined to be a key predictor of having a primary healthcare provider in our sample. Based on findings from the present study, TNB newcomers and non-permanent residents originating from highlighted regions and residing in Quebec or the Prairie provinces may benefit from settlement agency initiatives that connect them to physicians who are competent in cross-cultural trans health.

### Data availability statement

The 2019 Trans PULSE Canada data cannot be deposited in a data repository due to conditions in the letter of information and consent for participants that specified data would only be seen by members of the large national team's Data Analysis Working Group. These conditions were necessary in the context of stigmatization and distrust of researchers.

## Funding

This study was funded by the 10.13039/501100000024Canadian Institutes of Health Research (Funding Reference Number PJT-159690). Monica A. Ghabrial is funded by the 10.13039/501100000024Canadian Institutes of Health Research Fellowship (472688).

## CRediT authorship contribution statement

**Monica A. Ghabrial:** Writing – review & editing, Writing – original draft, Formal analysis, Conceptualization. **Tatiana Ferguson:** Writing – review & editing, Conceptualization. **Ayden I. Scheim:** Writing – review & editing, Methodology, Investigation, Funding acquisition, Data curation, Conceptualization. **Noah J. Adams:** Writing – review & editing, Conceptualization. **Moomtaz Khatoon:** Writing – review & editing, Conceptualization. **Greta R. Bauer:** Writing – review & editing, Supervision, Resources, Project administration, Methodology, Investigation, Funding acquisition, Data curation, Conceptualization.

## Declaration of competing interest

The authors declare the following financial interests/personal relationships which may be considered as potential competing interests:

Greta Bauer reports financial support was provided by Canadian Institutes of Health Research. Monica Ghabrial receives financial support from the Canadian Institutes of Health Research Fellowship. The other authors declare that they have no known competing financial interests or personal relationships that could have appeared to influence the work reported in this paper.
